# Molecular and clinical characterization of PARP9 in gliomas: A potential immunotherapeutic target

**DOI:** 10.1111/cns.13380

**Published:** 2020-04-24

**Authors:** Hao Xu, Songshan Chai, Yihao Wang, Jiajing Wang, Dongdong Xiao, Junjun Li, Nanxiang Xiong

**Affiliations:** ^1^ Department of Neurosurgery Union Hospital Tongji Medical College Huazhong University of Science and Technology Wuhan China

**Keywords:** biomarker, glioma, immunotherapeutic targets, PARP9, prognosis

## Abstract

**Background:**

Glioma is a primary malignancy of the central nervous system (CNS). As biomedicine advances, an efficient molecular target is urgently needed for the diagnosis and treatment of glioma. Meanwhile, several studies have demonstrated that glioma development is closely related to immunity. PARP9 is an inactive mono‐ADP‐ribosyltransferase belonging to the poly‐ADP ribosyltransferase (ARTD) family. In this article, we aimed to reveal the relationship between PARP9 and glioma and explore the potential prognostic value and immunotherapeutic targetability of PARP9 in glioma.

**Methods:**

PARP9 transcript levels were analyzed with TCGA and GEO databases. The clinicopathological information of patients with glioma in the TCGA database and gene expression profiles were analyzed to determine the relationship between the expression of PARP9 and clinicopathologic characteristics. Kaplan‐Meier survival analysis, univariate Cox regression analysis, and multivariate Cox regression analysis were used for survival analysis. Gene set enrichment analysis (GSEA) and gene set variation analysis (GSVA) were used for bioinformatics analysis. Correlation analysis explored the relationships between PARP9, infiltrating inflammatory immune cells, and immune checkpoint molecules.

**Results:**

PARP9 is highly expressed in glioma, and high expression of PARP9 is associated with poor prognosis and advanced clinicopathological features. Bioinformatics analysis showed that some immune‐related pathways were closely associated with high expression of PARP9. Correlation analysis indicated that PARP9 was closely related to inflammatory and immune responses, high immune cell infiltration, and immune checkpoint molecules.

**Conclusions:**

PARP9 may serve as an unfavorable prognosis predictor for glioma and a potential immunotherapeutic target.

## 1 INTRODUCTION

Glioma is a primary malignancy of the central nervous system (CNS), accounting for 80% of all CNS malignancies.^1^ According to their histological features, gliomas are classified into astrocytomas, ependymomas, oligodendrogliomas, mixed gliomas, and brainstem gliomas.^2^ Despite advances in a variety of treatments, overall survival (OS) improvements in glioma patients have been limited.^3^ With advances in biomedical technology, a variety of biomarkers and molecular classifications of glioma have been established. However, most of the markers are of limited use in the diagnosis and treatment of glioma. Therefore, it is urgent to identify effective markers and therapeutic targets for glioma.

Immune checkpoint inhibitors (ICIs) perform well in the treatment of solid tumors, including melanoma,^4^ non–small‐cell lung cancer,^5^ and renal cell carcinoma.^6^ Moreover, the discovery of lymphatic vessels in the central nervous system makes the immunotherapy of glioma feasible.^7^ However, immunotherapy currently does not work well for most gliomas. In previous studies, PD‐L1 (programmed death ligand 1),^8^ TIM3 (T‐cell immunoglobulin mucin‐3),^9^ and IDO1 (indoleamine 2,3‐dioxygenase 1)^10^ transcript levels were strongly correlated with immune responses and prognosis in gliomas. A large amount of research has led to a growing recognition of the relationship between glioma and immunity.

PARP9 is an inactive mono‐ADP‐ribosyltransferase belonging to the intracellular diphtheria toxin‐like glutamate/aspartate‐specific mono‐ and poly‐ADP ribosyltransferase (ARTD) family (also known as PARPs).^11^ However, PARP9 lacks PARP activity, despite possessing carboxy‐terminal amino acid sequences similar to those of other members of the PARP family.^12^ Previous studies have shown that PARP9 is overexpressed in a series of solid tumors, such as breast tumors,^12^ prostate tumors,^11^ diffuse large B cell lymphomas,^13^ and cervical tumors,^14^ and PARP9 may promote metastasis, recurrence and chemotherapy resistance in these tumors. Previous studies have also shown that PARP9 can regulate macrophages,^15^ which are the major type of immune cell within brain tumors, often comprising up to 30% of the tumor mass,^16^ indicating that PARP9 may influence tumor immune infiltration in glioma. However, the expression of PARP9, its clinical significance and its relationship with immune infiltration in glioma remain elusive. Thus, this study aimed to reveal the relationship between PARP9 and glioma and explore the potential prognostic value and immunotherapeutic targetability of PARP9 in glioma.

## 2 METHOD

### 2.1 Gene expression profile data and clinical data analysis

Microarray data of glioma patients were obtained from the Gene Expression Ominibus (GEO)^17^ under the accession number GSE50161^18^; the GPL570 platform (Affymetrix Human Genome U133 Plus 2.0 Array) was used. The RNA‐seq datasets and clinical data from patients with glioma from The Cancer Genome Atla (TCGA) (https://cancergenome.nih.gov/)^19^ were downloaded and analyzed. The TCGA glioma datasets comprised low‐grade glioma (LGG) and GBM datasets (HTSeq‐FPKM), and they were further analyzed for associations between PARP9 expression, clinicopathologic characteristics and immune cell infiltration.

### 2.2 Bioinformatics analysis

Gene set enrichment analysis (GSEA) was performed to identify differentially enriched biological pathways between the high PARP9 expression and low PARP9 expression groups. In addition, gene set variation analysis (GSVA) was performed to transform the gene expression patterns of all samples in the TCGA database into scores for inflammatory response metagenes, and correlograms were used to further verify the correlations between PARP9 and these metagenes.

### 2.3 Statistical analysis

In this study, SPSS 25.0, R software 3.6 and GraphPad Prism 7.0 statistical software were used to conduct the analysis. The variables distribution was checked by the Shapiro‐Wilk test. Student's *t*‐test was performed to evaluate data that follows a normal distribution, for other variables the Mann‐Whitney test was used. Descriptive statistics were used to summarize the molecular and clinical information from the TCGA database. Logistic regression tests were used to analyze the relationship between PARP9 and clinicopathological features. Kaplan‐Meier survival analysis, univariate Cox regression analysis, and multivariate Cox regression analysis were used to compare the effects of PARP9 expression and other clinical variables on overall survival in patients. Classical correlation analysis was used to detect the correlation between PARP9 expression, inflammatory type, immune cell type, and immune checkpoint molecules. The heat map, circos, and corrgram functions were conducted in the R software. A *P* value <.05 was considered statistically significant.

## 3 RESULTS

### 3.1 Expression of PARP9 in glioma and normal samples

**FIGURE 1:**
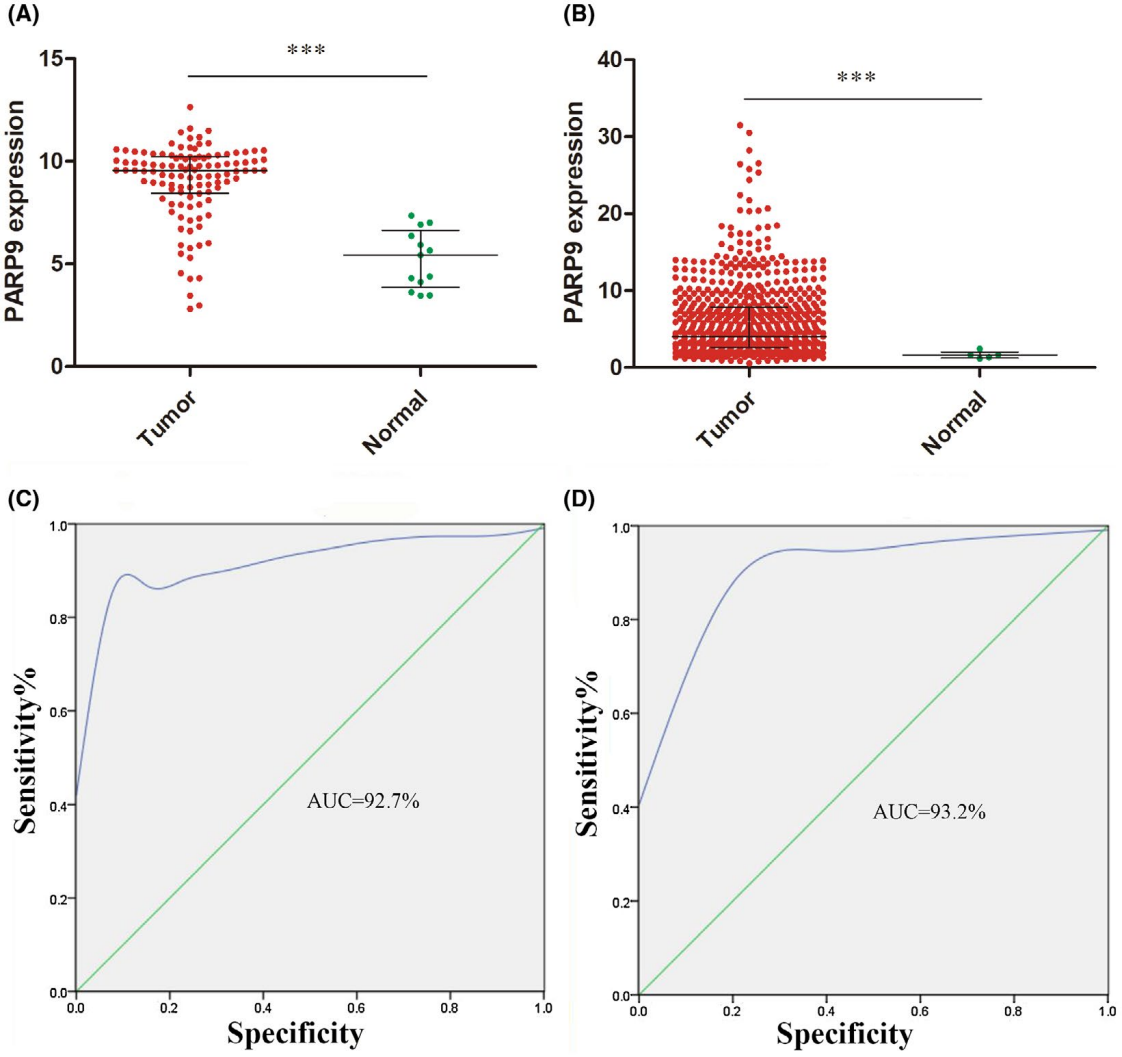
A and B, PARP9 was highly expressed in glioma for GSE50161 from GEO database and TCGA database. C and D, ROC curve analysis revealed the predictive value of PARP9 in diagnosis of glioma for GSE50161 from GEO database and TCGA database

By analyzing the RNA sequencing data of glioma and normal brain tissue from the TCGA database, we found that PARP9 was significantly upregulated in glioma compared to nontumor tissues (Figure 1A, *P* < .001). Moreover, we also obtained the GSE50161 dataset from the GEO database to verify the results (Figure 1B, *P* < .001). In addition, ROC curves were generated based on PARP9 expression and sample type for two datasets. As shown in Figure 1C,D, the area under the curve (AUC) reached 92.7% and 93.2% in the GEO and TCGA datasets, respectively. These results suggested that PARP9 may be a potential biomarker in glioma.

### 3.2 Glioma patient characteristics

The analysis of the relationship between PARP9 expression and clinicopathological features was performed with data from the TCGA database. A total of 1,114 cases contained 194 astrocytomas, 191 oligodendrogliomas, 130 oligoastrocytomas, 596 GBMs, and 3 cases without histological information. The average age overall was 52 years, and the cases included 651 males and 460 females. In terms of the grade of the tumor, 249 grade II, 265 grade III, and 596 grade IV tumors were included in our cohort. In terms of IDH mutations, 125 cases had mutation information. Of these, 91 (72.8%) cases were mutated, and 34 (27.2%) cases were wild type. In terms of the KPS (Karnofsky performance score), 584 (52.4%) patients scored ≥80 points, and 151 (13.6%) patients scored <80 points. In terms of tumor status, 209 (18.8%) patients were tumor‐free, and 783 patients (70.3%) had tumors. Overall, 570 (51.7%) patients were alive and 539 (48.3%) patients had died at the last follow‐up (Table 1).

**TABLE 1:** Basic glioma patient information

Clinical information	No. Of patients (%)
Age at diagnosis	
Median (range)	52 (9‐89)
Gender	
Men	651 (58.4)
Women	460 (41.3)
Unknow	3 (0.27)
WHO grade	
G2	249 (22.4)
G3	265 (23.8)
G4	596 (53.5)
Unknow	4 (3.6)
Histological type	
Astrocytomas	196 (17.4)
Oligodendrogliomas	191 (17.2)
Oligoastrocytomas	130 (11.7)
Glioblastoma	596 (53.5)
Unknow	4 (0.27)
IDH mutation	
YES	91 (8.17)
NO	34 (3.05)
Unknow	989 (88.8)
KPS	
<80	151 (13.6)
≥80	584 (52.4)
Unknow	379 (34.0)
Tumor status	
Tumor free	209 (18.8)
With tumor	783 (70.3)
Unknow	122 (11.0)
Vital status	
Alive	570 (51.2)
Dead	539 (48.4)
Unknow	5 (0.45)

### 3.3 Relationship between PARP9 expression and clinicopathological characteristics

**FIGURE 2:**
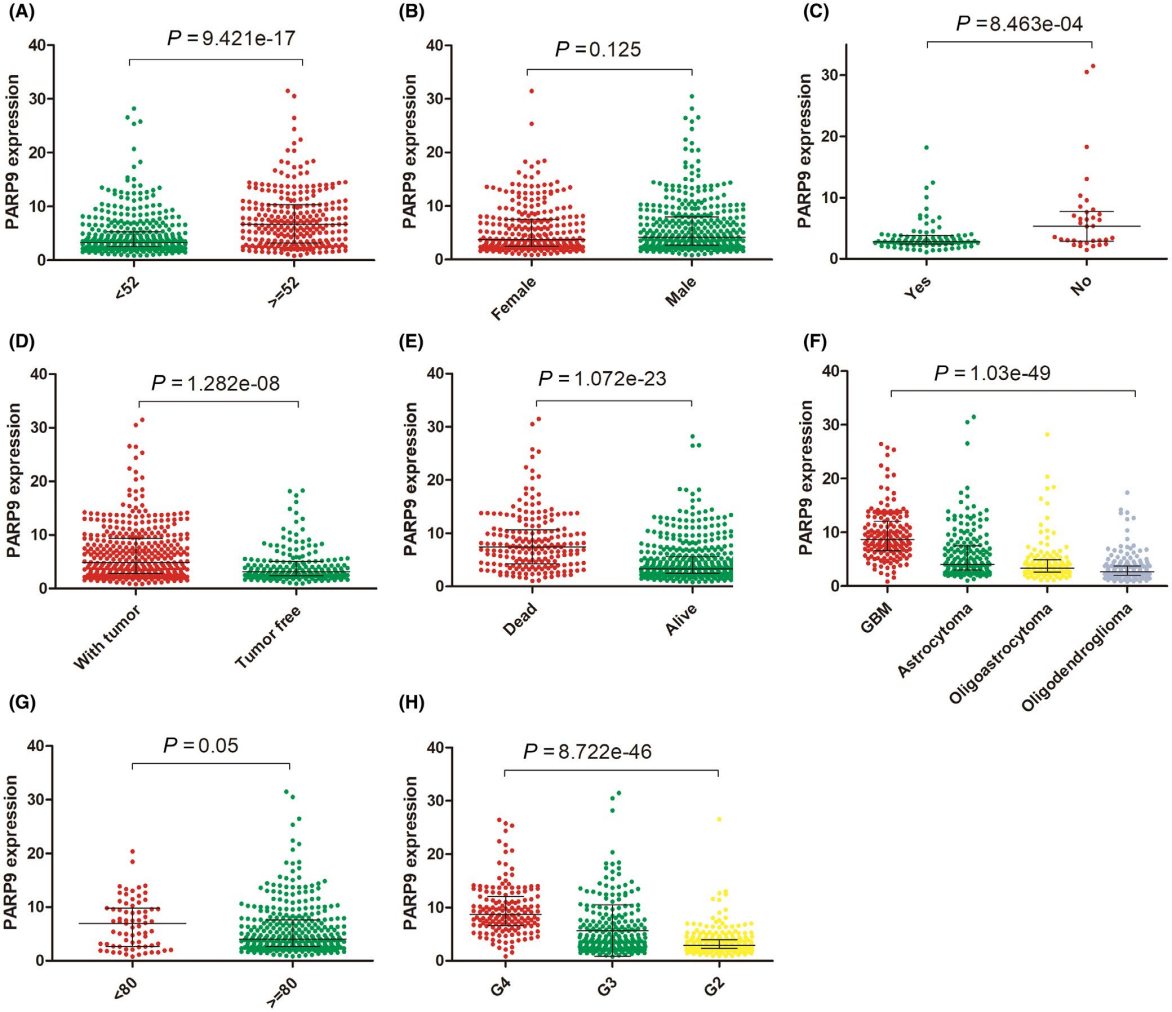
Relationship between the expression of PARP9 and clinicopathologic features in TCGA cohort. (A): age, (B): gender, (C): IDH mutation, (D): tumor status. (E): vital status, (F): histological type, (G): KPS, (H): grade

PARP9 expression and clinicopathological characteristics were compared for analysis to explore the features related to PARP9 expression. As shown in Figure 2, PARP9 expression was significantly related to age (*P* < .001), tumor grade (*P* < .001), histological type (*P* < .001), IDH mutation (*P* < .001), KPS (*P* = .05), tumor status (*P* < .001), and vital status (*P* < .001).

Logistic regression analysis revealed that increased PARP9 was associated with poor prognosis (Table 2). High PARP9 expression was linked to age (≥52 years vs < 52 years, OR = 3.52, 95% Cl 2.54‐4.91, *P* < .001), histological type (GBM vs oligoastrocytoma, OR = 21.83, 95% Cl 11.15‐46.51, *P* < .001; GBM vs astrocytoma, OR = 13.26, 95% Cl 7.02‐27.40, *P* < .001; GBM vs oligodendroglioma, OR = 50.79, 95% Cl 26.10‐107.95, *P* < .001), grade (G3 vs G2, OR = 2.57, 95% Cl 1.78‐3.77, *P* < .001; G4 vs G2, OR = 39.77, 95% Cl 21.07‐82.32, *P* < .001), IDH mutation (no vs yes, OR = 3.35, 95% Cl 1.47‐8.10, *P* < .05), tumor status (tumor vs tumor‐free, OR = 2.37, 95% CI 1.67‐3.40, *P* < .001), and KPS (<80 vs ≥ 80, OR = 2.08, 95% CI 1.23‐3.58, *P* < .05). Collectively, these data suggest that PARP9 may serve as an oncogene for glioma and can promote poor prognosis.

**TABLE 2:** Association between PARP9 expression and clinicopathologic variables using logistic regression

Clinical feature	Odds ratio in PARP9 expression	*P*‐value
Age		
≥52 vs < 52	3.52 (2.54‐4.91)	<.001
Gender		
Man vs Women	1.12 (0.93‐1.48)	.58
WHO grade		
Ⅲ vs Ⅱ	2.57 (1.78‐3.77)	<.001
Ⅳ vs Ⅱ	39.77 (21.07‐82.32)	<.001
Histological type		
GBM vs Astrocytoma	13.26 (7.02‐27.40)	<.001
GBM vs Oligodendroglioma	50.79 (26.10‐107.95)	<.001
GBM vs Oligoastrocytomas	21.83 (11.15‐46.51)	<0.001
IDH mutation		
No vs Yes	3.35 (1.47‐8.10)	.005
KPS		
<80 vs ≥ 80	2.08 (1.23‐3.58)	.007
Tumor status		
With tumor vs Tumor free	2.37 (1.67‐3.40)	<.001
Vital status		
Dead vs Alive	5.58 (3.84‐8.24)	<.001

### 3.4 PARP9 predicted poor prognosis in glioma

**FIGURE 3:**
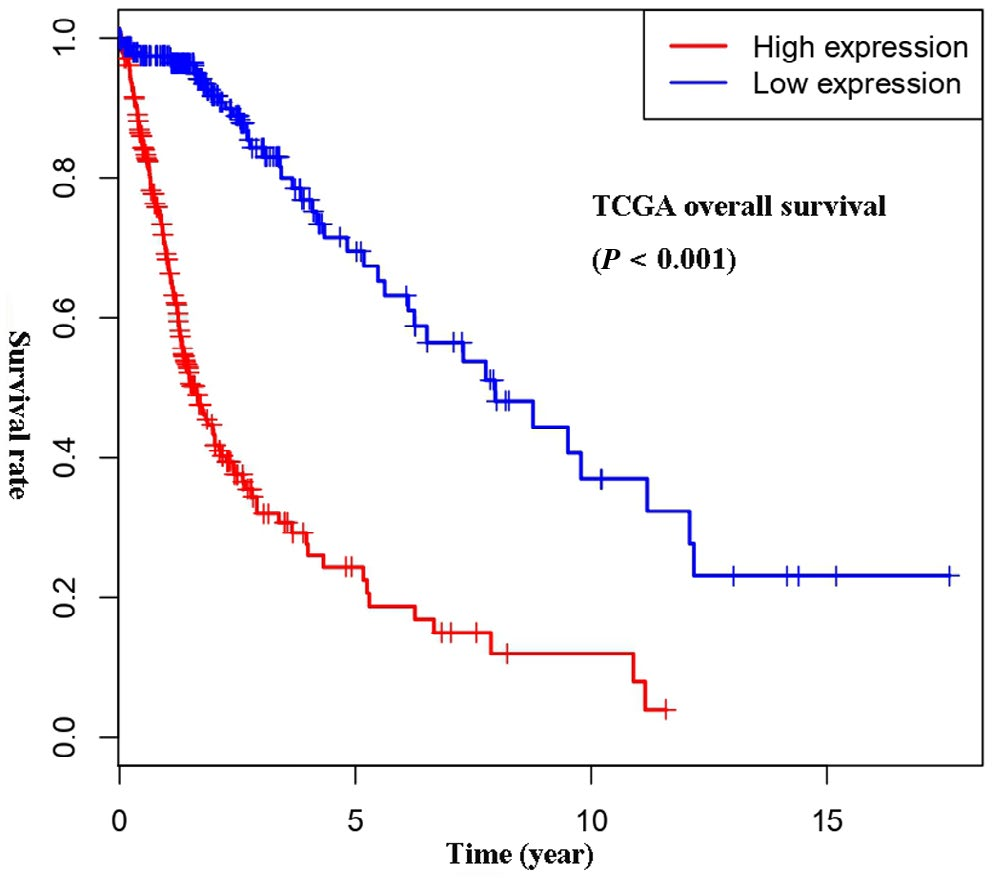
Survival analysis of PARP9 in all‐grade glioma in the TCGA cohort

To analyze the prognostic value of PARP9 in glioma, Kaplan‐Meier curves were constructed with data from the TCGA database. As shown in Figure 3, patients with high PARP9 expression had significantly poorer survival than patients with low PARP9 expression (*P* < .001). The results indicated that PARP9 was a poor prognosis marker in glioma (Table 3).

**TABLE 3:** Univariate analysis of over survival in TCGA

Clinical characteristics	HR (95% CI)	*P*‐value
Age	1.07 (1.05‐1.08)	<.001
Gender	0.98 (0.70‐1.38)	.92
WHO grade	4.66 (3.48‐6.25)	<.001
Histological type	2.45 (2.00‐3.00)	<.001
KPS	0.95 (0.94‐0.96)	<.001
Tumor status	37.06 (5.18‐265.21)	<.001
PARP9	1.10 (1.07‐1.12)	<.001

Univariate and multivariate Cox analyses were performed to further explore the prognostic value of PARP9 in glioma. Cases with complete information were selected, and 371 cases were studied. Univariate Cox regression analysis showed that PARP9 was significantly correlated with survival (HR = 1.10, 95% CI 1.07‐1.12, *P* < .001). Other factors, such as age (HR = 1.07, 95% CI 1.05‐1.08 *P* < .001), KPS (HR = 0.95, 95% CI 0.94‐0.96, *P* < .001), tumor status (HR = 37.06, 95% CI 5.18‐265.21, *P* < .001), WHO grade (HR = 4.66, 95% CI 3.48‐6.25, *P* < .001), and histological type (HR = 2.45, 95% CI 2.00‐3.00, *P* < .001), were also significantly related to survival. In the multivariate analysis, high PARP9 expression in patients was independently associated with overall survival (HR = 1.04, 95% CI 1.01‐1.07, *P* = .009), together with tumor status (HR = 23.64, 95% CI 3.29‐169.85, *P* = .002) and histological type (HR = 2.02, 95% CI 1.63‐2.49, *P* < .001). The above results indicated that PARP9 might be a new independent prognostic molecular marker for glioma patients.

### 3.5 PARP9‐related biological process and signaling pathways

**FIGURE 4:**
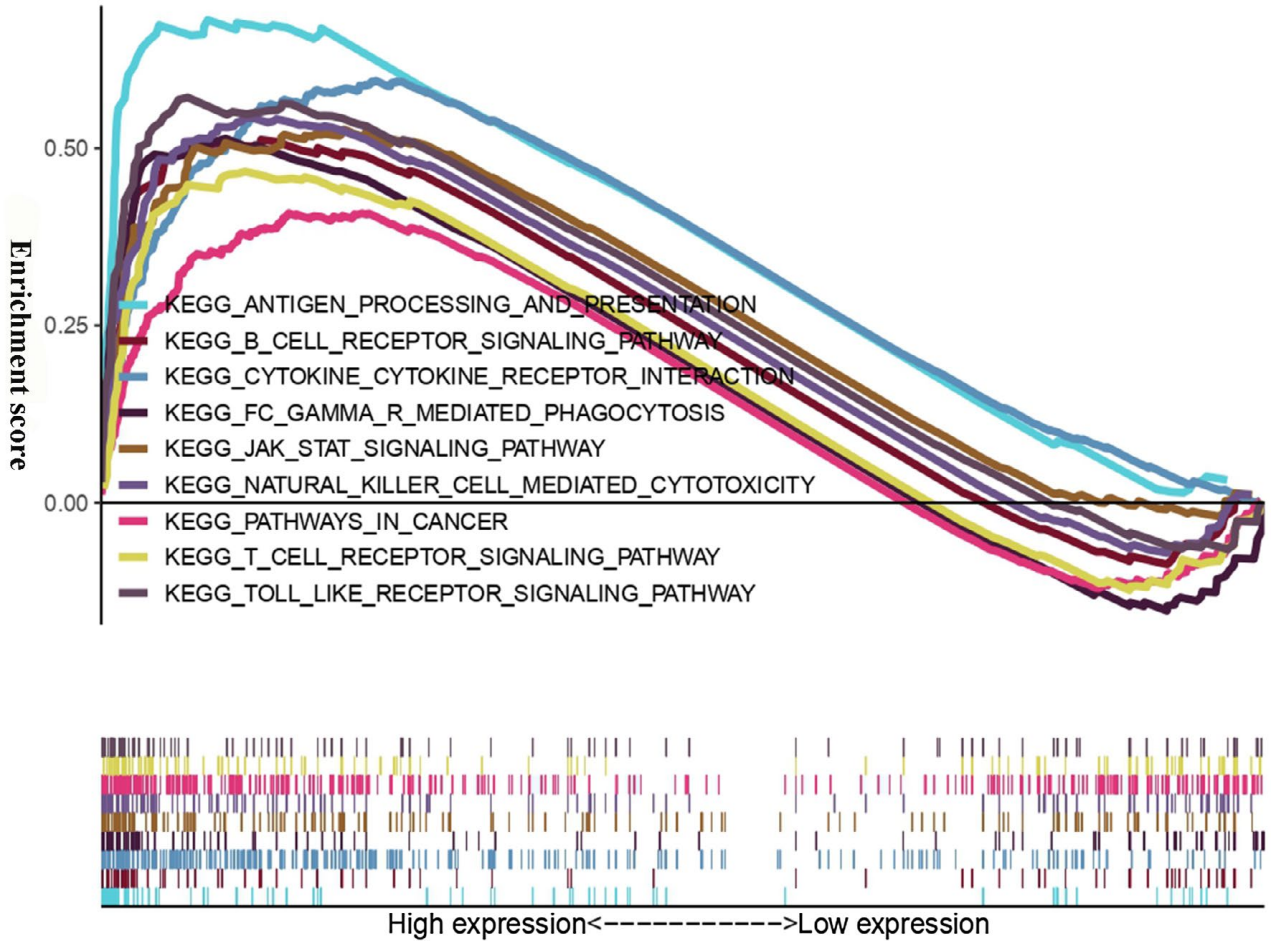
Gene set enrichment analysis (GSEA) for PARP9

We explored and verified the biological processes and signaling pathways associated with PARP9 expression using gene set enrichment analysis (GSEA). As shown in Figure 4 and Table 4, several biological processes and signaling pathways were enriched in patients with high PARP9 expression, such as antigen processing and presentation, the B cell receptor signaling pathway, cytokine‐cytokine receptor interactions, Fc gamma R‐mediated phagocytosis, the JAK‐STAT signaling pathway, natural killer cell‐mediated cytotoxicity, pathways in cancer, the T‐cell receptor signaling pathway and the Toll‐like receptor signaling pathway. Most of these biological processes and signaling pathways are involved in immune and inflammatory responses.

**TABLE 4:** Multivariate analysis of over survival in TCGA

Clinical characteristics	HR (95% CI)	*P*‐value
Histological type	2.02 (1.63‐2.49)	<.001
Tumor status	23.64 (3.29‐169.85)	<.001
PARP9	1.04 (1.01‐1.17)	.009

### 3.6 Correlation between PARP9 and inflammatory activities

**FIGURE 5:**
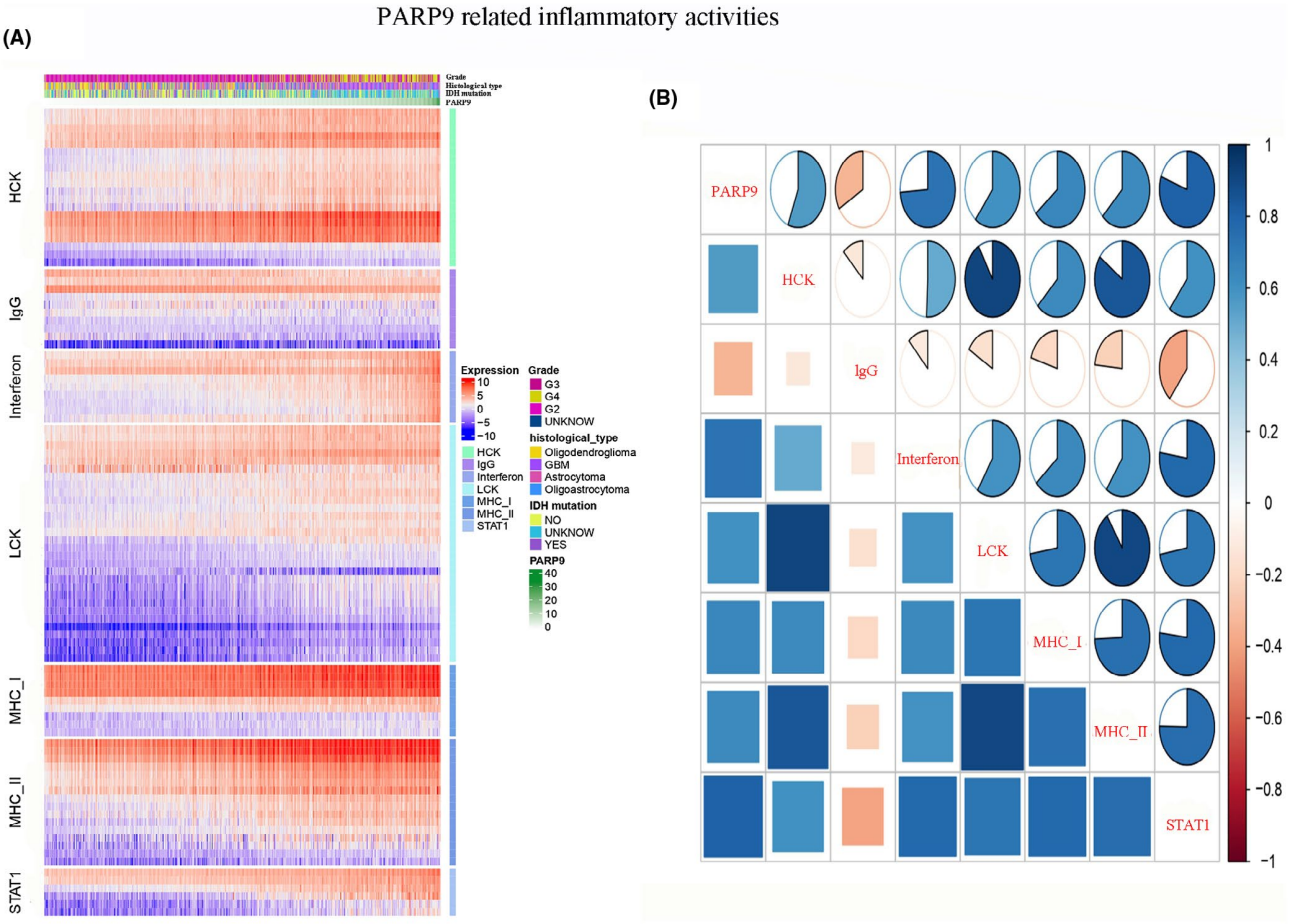
PARP9‐related inflammatory response. A, Heat map involved the clinicopathological features, PARP9 expression, and seven related metagenes from the TCGA datasets. B, Correlograms were generated based on the relationship between PARP9 expression and GSVA enrichment scores for these seven metagenes. The circles were filled in blue clockwise for positive values and in red anticlockwise for negative values

To thoroughly understand PARP9‐related inflammatory activities, seven clusters containing 104 genes (File S1) representing different types of inflammation and immune activities were used for study.^20^ As shown in Figure 5A, the expression of PARP9 was positively related to most of the gene clusters, such as the HCK, LCK, interferon, STAT1, MHC I, and MHC II clusters, yet it was negatively correlated with the IgG cluster, which represented B cells. To verify our above analysis, GSVA was performed to convert gene expression data into enrichment scores for metagenes. Correlograms were generated to visualize the relationship between PARP9 and seven metagenes, and the result was consistent with our above findings (Figure 5B; Table 5).

**TABLE 5:** Gene sets enriched in the high expression phenotype group

Gene set name	NES	NOM *P*‐value	FDR q‐value
KEGG_ANTIGEN_PROCESSING_AND_PRESENTATION	2.07	.002	0.0056
KEGG_B_CELL_RECEPTOR_SIGNALING_PATHWAY	1.75	.020	0.0347
KEGG_CYTOKINE_CYTOKINE_RECEPTOR_INTERACTION	2.02		0.0051
KEGG_FC_GAMMA_R_MEDIATED_PHAGOCYTOSIS	1.89	.010	0.0105
KEGG_JAK_STAT_SIGNALING_PATHWAY	1.93	.002	0.0079
KEGG_NATURAL_KILLER_CELL_MEDIATED_CYTOTOXICITY	1.98		0.0056
KEGG_PATHWAYS_IN_CANCER	1.68	.012	0.0483
KEGG_T_CELL_RECEPTOR_SIGNALING_PATHWAY	1.71	.008	0.0445
KEGG_TOLL_LIKE_RECEPTOR_SIGNALING_PATHWAY	1.98		0.0051

Abbreviations: FDR q‐val, false discovery rate q‐value; NES, normalized enrichment score; NOM *P*‐val, normalized *P*‐value.

### 3.7 Correlation of PARP9 with infiltrating immune cells

**FIGURE 6:**
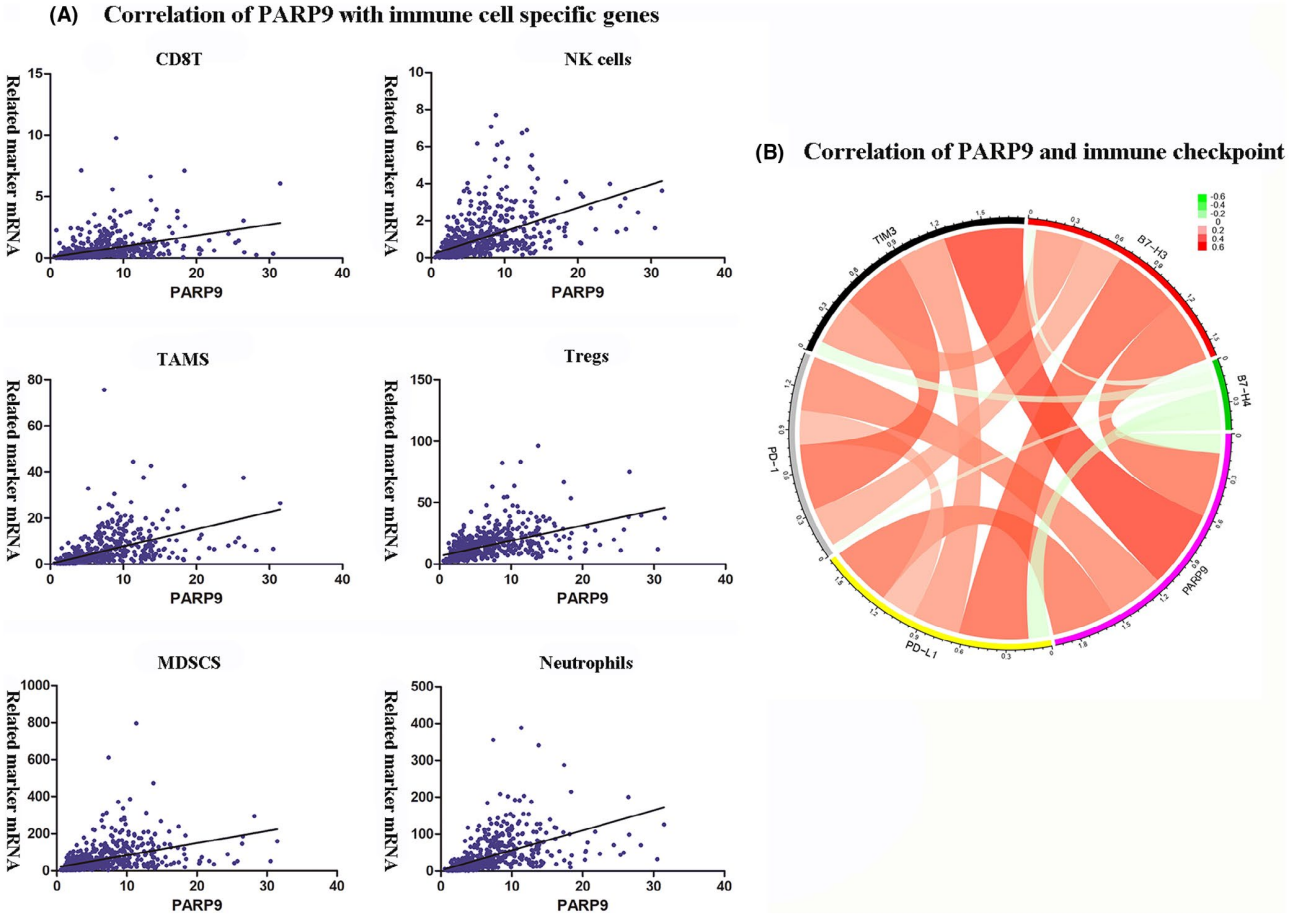
A, Correlations of PARP9 expression with immune cell markers. B, Relationship between PARP9 and immune checkpoints in the TCGA databases

Previous research has shown that tumor‐infiltrating immune cells play a critical role in regulating tumor progression and prognosis.^10^, ^21^, ^22^ We investigated the relationship between the expression of PARP9 and six immune cells that frequently infiltrate the tumor, including CD8+T cells, natural killer (NK) cells, tumor‐associated macrophages (TAMs), regulatory T cells (Tregs), myeloid‐derived suppressor cells (MDSCs), and neutrophils. The specific markers used for the immune cells are listed in File S2. Correlation analysis showed that PARP9 expression was positively correlated with 6 immune cell‐specific markers, which indicated that patients with higher PARP9 expression were more likely to have more infiltrating immune cells than patients with lower PARP9 expression in glioma (Figure 6A).

### 3.8 PARP9 correlates with immune checkpoint molecules in the tumor‐directed immune response

Five immune checkpoint molecules were evaluated as therapeutic targets: PD‐1, PD‐L1, B7‐H3, B7‐H4, and TIM‐3.^23^, ^24^, ^25^ The relationship between PARP9 and five immune checkpoint molecules in TCGA data was studied by Pearson correlation analysis. The results showed that PARP9 had a strong positive relationship with PD‐1 and PD‐L1, suggesting that it is closely related to the PD‐1/PD‐L1 pathway (Figure 6B). Likewise, B7‐H3 and TIM‐3 were also strongly related to PARP9 expression. These results indicated that PARP9 was closely related to common immunotherapeutic targets and that PARP9 may be a new immunotherapeutic target.

## 4 DISCUSSION

In this study, the expression of PARP9 was identified to be upregulated in glioma samples using the TCGA and GEO databases. We retrospectively analyzed 1,114 glioma patients from the TCGA RNA‐seq dataset. It was determined that PARP9 significantly predicted overall survival in all‐grade gliomas and GBMs. Moreover, the PARP9 expression level increased with glioma grade and was associated with clinicopathological parameters (age, vital status, tumor status, histological type, KPS, and IDH1 status). A Cox regression analysis with univariate and multivariate analyses demonstrated that PARP9 may be a valuable prognostic biomarker for glioma patients. All these results indicate that the expression of PARP9 is closely correlated with the development and malignant progression of glioma.

Using GSEA, gene sets related to inflammatory and immune responses were found to be enriched in patients with high PARP9 expression. To better understand PARP9‐related inflammatory activities, heat maps involving seven clusters of 104 genes were generated and revealed that PARP9 was positively correlated with for the HCK, interferon, LCK, MHC I, MHC II, and STAT1 metagenes but negatively correlated with the IgG metagene, a marker for B cells. Correlogram analysis using GSVA verified the abovementioned results. Canonical correlation analysis revealed significant positive correlations between PARP9 expression and the gene expression of specific markers of six crucial immune cells, consisting of adaptive (Tregs and CD8+T cells) and innate (NK cells, TAMs, MDSCs, and neutrophils) immune cells. Additionally, circos plots demonstrated that PARP9 tightly correlated with changes in the levels of several prominent immune checkpoint molecules. These findings suggest that PARP9 plays an important role in the glioma immune microenvironment.

Over the past decade, high‐throughput sequencing has been widely used to understand the molecular mechanisms underlying the progression of disease.^26^ Recently, several studies have shown that PARP9 exhibits altered expression in various human tumors. Knockdown of PARP9 was indicated to inhibit the migration of breast cancer cells, which suggested that PARP9 may promote breast cancer progression.^12^ PARP9 has been shown to contribute to tumor recurrence, metastasis, and resistance to chemotherapy in prostate cancer.^11^ Furthermore, in high‐risk diffuse large B cell lymphoma, PARP9 might be related to lymphocyte migration and promote malignant B cell dissemination.^27^, ^28^ Recently, Su et al demonstrated that the TME‐related eight‐gene signature, including PARP9, was significantly associated with the prognosis of lower‐grade gliomas patients, which supported our results.^29^ In our study, we firstly performed the comprehensive research to identify the expression pattern and distribution of PARP9 in 1,114 glioma samples, which include, but are not limited to, lower‐grade gliomas. Increased PARP9 expression was found to be significantly related to advanced clinicopathologic characteristics and poor prognosis in glioma patients. These results suggested that PARP9 may be a valuable therapeutic target for glioma gene therapy.

In previous studies, glioma cells were found to be able to produce numerous cytokines, which contribute to the infiltration of different types of immune cells, such as CD4+T cells, CD8+T cells, Tregs, NK cells, TAMs, MDSCs, and neutrophils, into the tumor.^30^ These nonneoplastic cells create a tumor microenvironment that plays an essential role in tumor growth, recurrence, metastasis, and response to therapeutic intervention. Although the immune system is clearly capable of generating immune responses directed against tumor cells, these responses are insufficient to eradicate tumors in most patients because of local immunosuppression within the tumor microenvironment.^31^ Therefore, overcoming the immunosuppressive state is critical to improving the effectiveness of anticancer immunotherapeutic treatments. An interesting and novel finding in our study was that PARP9 was involved in the glioma immune microenvironment. A significant positive correlation was observed between PARP9 transcriptional level and immunosuppressive cells, including Tregs, MDSCs, and neutrophils. These cells can exhibit potent immunosuppressive activities and result in adverse prognoses for cancer patients. Certainly, future research is required to elucidate the detailed interactions of PARP9 with immunosuppressive cells.

During the last few years, remarkable advances have been made in the field of cancer immunotherapy, largely driven by the considerable success of ICIs in other tumor types.^32^, ^33^ However, glioma has remained largely refractory to current immunotherapies.^34^, ^35^ Enhanced response rates to a combination of two different ICIs have been reported in patients with melanomas compared with the responses seen with either agent as monotherapy.^36^ Previous studies have reported that PD‐L1, TIM‐3, and IDO1 transcriptional levels are highly associated with immune response and prognosis in patients with glioma.^8^, ^9^, ^10^ Furthermore, experimental results have revealed that combination anti‐PD‐1 plus anti‐TIM‐3 treatment can achieve a longer overall survival than anti‐TIM‐3 monotherapy in murine gliomas.^37^ Targeting TAMs via the inhibition of the colony‐stimulating factor‐1 receptor (CSF‐1R) pathway has emerged as an attractive approach for anticancer therapy.^38^ Dual PD‐1 and CSF‐1R blockade could promote antitumor activity in preclinical models.^39^, ^40^ Encouragingly, a clinical trial has reported that the combination of anti‐PD‐1 and anti‐CSF‐1R therapies may provide a durable clinical benefit for malignant glioma patients (ClinicalTrials.gov identifier: NCT02526017). These exciting results have sparked increasing interest in discovering new immunotherapeutic targets for the treatment of glioma. In the present study, we identified PARP9 as a potential immunotherapeutic target. As seen in Figure 6, PARP9 had a high concordance with prominent immune checkpoint molecules, including PD‐1, PD‐L1, B7‐H3, and TIM‐3, suggesting their synergistic roles in regulating the immune response within the tumor microenvironment. These findings open up new possibilities for combination therapy in glioma. The combination of PARP9 and immune checkpoints inhibitors may help to overcome the limitations related to the administration of immune checkpoints inhibitors alone.

Our study has several limitations. First, a low number of patients were included for the univariate and multivariate Cox analyses due to the lack of complete information about all variables in some of the patients. Second, owing to the small number of negative samples serving as controls, additional studies are required. Third, it is generally known that the expression of mRNA does not always predict protein levels because the transcription process can be changed in tumors.^41^ Therefore, to better understand the essential role of PARP9, further study of PARP9 protein levels in glioma samples is necessary. Certainly, other laboratory research should also be undertaken to expound the exact mechanism of PARP9 overexpression in gliomas and explain its correlation with immunomodulation and poor outcome in gliomas.

## 5 CONCLUSIONS

In summary, this study demonstrates that PARP9 is overexpressed in glioma samples. High PARP9 expression is associated with advanced clinicopathological parameters and predicts much worse survival for glioma patients. We also found that PARP9 was involved in the inflammatory and immune responses and was correlated with checkpoint molecules. Therefore, taken together, PARP9 may serve as an unfavorable prognosis predictor for glioma and a potential immunotherapeutic target, which, when used in combination, may improve the therapeutic efficacy of ICIs.

## CONFLICT OF INTEREST

The authors declare no conflict of interest.

## Supporting information

File S1

File S2

## References

[1] Omuro A , DeAngelis LM . Glioblastoma and other malignant gliomas: a clinical review. JAMA. 2013;310:1842‐1850. 24193082 10.1001/jama.2013.280319

[2] Morgan LL . The epidemiology of glioma in adults: a "state of the science" review. Neuro Oncol. 2015;17:623‐624. 25605816 10.1093/neuonc/nou358 PMC4483082

[3] Woehrer A , Bauchet L , Barnholtz‐Sloan JS . Glioblastoma survival: has it improved? Evidence from population‐based studies. Curr Opin Neurol. 2014;27:666‐674. 25364955 10.1097/WCO.0000000000000144

[4] Kato K , Cho BC , Takahashi M , et al. Nivolumab versus chemotherapy in patients with advanced oesophageal squamous cell carcinoma refractory or intolerant to previous chemotherapy (ATTRACTION‐3): a multicentre, randomised, open‐label, phase 3 trial. Lancet Oncol. 2019;20:1506‐1517. 31582355 10.1016/S1470-2045(19)30626-6

[5] Gandhi L , Rodriguez‐Abreu D , Gadgeel S , et al. Pembrolizumab plus Chemotherapy in Metastatic Non‐Small‐Cell Lung Cancer. N Engl J Med. 2018;378:2078‐2092. 29658856 10.1056/NEJMoa1801005

[6] Gettinger SN , Horn L , Gandhi L , et al. Overall Survival and Long‐Term Safety of Nivolumab (Anti‐Programmed Death 1 Antibody, BMS‐936558, ONO‐4538) in Patients With Previously Treated Advanced Non‐Small‐Cell Lung Cancer. J Clin Oncol. 2015;33:2004‐2012. 25897158 10.1200/JCO.2014.58.3708 PMC4672027

[7] Louveau A , Smirnov I , Keyes TJ , et al. Corrigendum: Structural and functional features of central nervous system lymphatic vessels. Nature. 2016;533:278. 10.1038/nature16999 26909581

[8] Wang Z , Zhang C , Liu X , et al. Molecular and clinical characterization of PD‐L1 expression at transcriptional level via 976 samples of brain glioma. Oncoimmunology. 2016;5:e1196310. 27999734 10.1080/2162402X.2016.1196310 PMC5139638

[9] Li G , Wang Z , Zhang C , et al. Molecular and clinical characterization of TIM‐3 in glioma through 1,024 samples. Oncoimmunology. 2017;6:e1328339. 28919992 10.1080/2162402X.2017.1328339 PMC5593703

[10] Zhai L , Ladomersky E , Lauing KL , et al. Infiltrating T Cells Increase IDO1 Expression in Glioblastoma and Contribute to Decreased Patient Survival. Clin Cancer Res. 2017;23:6650‐6660. 28751450 10.1158/1078-0432.CCR-17-0120 PMC5850948

[11] Bachmann SB , Frommel SC , Camicia R , et al. DTX3L and ARTD9 inhibit IRF1 expression and mediate in cooperation with ARTD8 survival and proliferation of metastatic prostate cancer cells. Mol Cancer. 2014;13:125. 24886089 10.1186/1476-4598-13-125 PMC4070648

[12] Tang X , Zhang H , Long Y , et al. PARP9 is overexpressed in human breast cancer and promotes cancer cell migration. Oncol Lett. 2018;16:4073‐4077. 30128030 10.3892/ol.2018.9124 PMC6096171

[13] Camicia R , Bachmann SB , Winkler HC , et al. BAL1/ARTD9 represses the anti‐proliferative and pro‐apoptotic IFNgamma‐STAT1‐IRF1‐p53 axis in diffuse large B‐cell lymphoma. J Cell Sci. 2013;126:1969‐1980. 23487038 10.1242/jcs.118174

[14] Tao L , Wang X , Zhou Q . Long noncoding RNA SNHG16 promotes the tumorigenicity of cervical cancer cells by recruiting transcriptional factor SPI1 to upregulate PARP9. Cell Biol Int. 2019. 10.1002/cbin.11272 31774223

[15] Iwata H , Goettsch C , Sharma A , et al. PARP9 and PARP14 cross‐regulate macrophage activation via STAT1 ADP‐ribosylation. Nat Commun. 2016;7:12849. 27796300 10.1038/ncomms12849 PMC5095532

[16] Liu S , Zhang C , Maimela NR , et al. Molecular and clinical characterization of CD163 expression via large‐scale analysis in glioma. OncoImmunology. 2019;8:1601478. 31143523 10.1080/2162402X.2019.1601478 PMC6527268

[17] Edgar R , Domrachev M , Lash AE . Gene Expression Omnibus: NCBI gene expression and hybridization array data repository. Nucleic Acids Res. 2002;30:207‐210. 11752295 10.1093/nar/30.1.207 PMC99122

[18] Griesinger AM , Birks DK , Donson AM , et al. Characterization of distinct immunophenotypes across pediatric brain tumor types. J Immunol. 2013;191:4880‐4888. 24078694 10.4049/jimmunol.1301966 PMC3827919

[19] The TCGA Legacy. Cell. 2018;173:281‐282. 29625044 10.1016/j.cell.2018.03.049

[20] Rody A , Holtrich U , Pusztai L , et al. T‐cell metagene predicts a favorable prognosis in estrogen receptor‐negative and HER2‐positive breast cancers. Breast Cancer Res. 2009;11:R15. 19272155 10.1186/bcr2234 PMC2688939

[21] Mantovani A , Marchesi F , Malesci A , et al. Tumour‐associated macrophages as treatment targets in oncology. Nat Rev Clin Oncol. 2017;14:399‐416. 28117416 10.1038/nrclinonc.2016.217 PMC5480600

[22] Domingues P , Gonzalez‐Tablas M , Otero A , et al. Tumor infiltrating immune cells in gliomas and meningiomas. Brain Behav Immun. 2016;53:1‐15. 26216710 10.1016/j.bbi.2015.07.019

[23] Romero D . Immunotherapy: PD‐1 says goodbye, TIM‐3 says hello. Nat Rev Clin Oncol. 2016;13:202‐203. 10.1038/nrclinonc.2016.40 26977783

[24] Koyama S , Akbay EA , Li YY , et al. Adaptive resistance to therapeutic PD‐1 blockade is associated with upregulation of alternative immune checkpoints. Nat Commun. 2016;7:10501. 26883990 10.1038/ncomms10501 PMC4757784

[25] Pardoll DM . The blockade of immune checkpoints in cancer immunotherapy. Nat Rev Cancer. 2012;12:252‐264. 22437870 10.1038/nrc3239 PMC4856023

[26] Doyle MA , Li J , Doig K , et al. Studying cancer genomics through next‐generation DNA sequencing and bioinformatics. Methods Mol Biol. 2014;1168:83‐98. 24870132 10.1007/978-1-4939-0847-9_6

[27] Juszczynski P , Kutok JL , Li C , et al. BAL1 and BBAP Are Regulated by a Gamma Interferon‐Responsive Bidirectional Promoter and Are Overexpressed in Diffuse Large B‐Cell Lymphomas with a Prominent Inflammatory Infiltrate. Mol Cell Biol. 2006;26:5348‐5359. 16809771 10.1128/MCB.02351-05 PMC1592708

[28] Aguiar RC , Yakushijin Y , Kharbanda S , et al. BAL is a novel risk‐related gene in diffuse large B‐cell lymphomas that enhances cellular migration. Blood. 2000;96:4328‐4334. 11110709

[29] Su J , Long W , Ma Q , et al. Identification of a Tumor Microenvironment‐Related Eight‐Gene Signature for Predicting Prognosis in Lower‐Grade Gliomas. Front Genet. 2019;10:1143. 31803233 10.3389/fgene.2019.01143 PMC6872675

[30] Gieryng A , Pszczolkowska D , Walentynowicz KA , Rajan WD , Kaminska B . Immune microenvironment of gliomas. Lab Invest. 2017;97(5):498‐518. 28287634 10.1038/labinvest.2017.19

[31] Rabinovich GA , Gabrilovich D , Sotomayor EM . Immunosuppressive strategies that are mediated by tumor cells. Annu Rev Immunol. 2007;25:267‐296. 17134371 10.1146/annurev.immunol.25.022106.141609 PMC2895922

[32] Hodi FS , Chiarion‐Sileni V , Gonzalez R , et al. Nivolumab plus ipilimumab or nivolumab alone versus ipilimumab alone in advanced melanoma (CheckMate 067): 4‐year outcomes of a multicentre, randomised, phase 3 trial. Lancet Oncol. 2018;19:1480‐1492. 30361170 10.1016/S1470-2045(18)30700-9

[33] Reck M , Rodríguez‐Abreu D , Robinson AG , et al. Pembrolizumab versus Chemotherapy for PD‐L1–Positive Non–Small‐Cell Lung Cancer. N Engl J Med. 2016;375:1823‐1833. 27718847 10.1056/NEJMoa1606774

[34] Huang J , Liu F , Liu Z , et al. Immune Checkpoint in Glioblastoma: Promising and Challenging. Front Pharmacol. 2017;8:242. 28536525 10.3389/fphar.2017.00242 PMC5422441

[35] Zhang X , Zhu S , Li T , et al. Targeting immune checkpoints in malignant glioma. Oncotarget. 2017;8:7157. 27756892 10.18632/oncotarget.12702 PMC5351697

[36] Hodi FSD , Chesney JP , Pavlick ACM , et al. Combined nivolumab and ipilimumab versus ipilimumab alone in patients with advanced melanoma: 2‐year overall survival outcomes in a multicentre, randomised, controlled, phase 2 trial. Lancet Oncol. 2016;17:1558‐1568. 27622997 10.1016/S1470-2045(16)30366-7 PMC5630525

[37] Kim JE , Patel MA , Mangraviti A , et al. Combination Therapy with Anti‐PD‐1, Anti‐TIM‐3, and Focal Radiation Results in Regression of Murine Gliomas. Clin Cancer Res. 2017;23:124‐136. 27358487 10.1158/1078-0432.CCR-15-1535 PMC5735836

[38] Ries CH , Cannarile MA , Hoves S , et al. Targeting Tumor‐Associated Macrophages with Anti‐CSF‐1R Antibody Reveals a Strategy for Cancer Therapy. Cancer Cell. 2014;25:846‐859. 24898549 10.1016/j.ccr.2014.05.016

[39] Zhu Y , Knolhoff BL , Meyer MA , et al. CSF1/CSF1R blockade reprograms tumor‐infiltrating macrophages and improves response to T‐cell checkpoint immunotherapy in pancreatic cancer models. Cancer Res. 2014;74:5057‐5069. 25082815 10.1158/0008-5472.CAN-13-3723 PMC4182950

[40] Peranzoni E , Lemoine J , Vimeux L , et al. Macrophages impede CD8 T cells from reaching tumor cells and limit the efficacy of anti‐PD‐1 treatment. Proc Natl Acad Sci U S A. 2018;115:E4041‐E4050. 29632196 10.1073/pnas.1720948115 PMC5924916

[41] Jiang Q , Crews LA , Holm F , et al. RNA editing‐dependent epitranscriptome diversity in cancer stem cells. Nat Rev Cancer. 2017;17:381‐392. 28416802 10.1038/nrc.2017.23 PMC5665169

